# Unravelling the Immunity of Poultry Against the Extracellular Protozoan Parasite *Histomonas meleagridis* Is a Cornerstone for Vaccine Development: A Review

**DOI:** 10.3389/fimmu.2018.02518

**Published:** 2018-11-02

**Authors:** Taniya Mitra, Fana Alem Kidane, Michael Hess, Dieter Liebhart

**Affiliations:** ^1^Clinic for Poultry and Fish Medicine, Department for Farm Animals and Veterinary Public Health, University of Veterinary Medicine Vienna, Vienna, Austria; ^2^Christian Doppler Laboratory for Innovative Poultry Vaccines (IPOV), University of Veterinary Medicine Vienna, Vienna, Austria

**Keywords:** *Histomonas meleagridis*, histomonosis, immunity, vaccination, immune response, extracellular parasite, poultry

## Abstract

The protozoan parasite *Histomonas meleagridis* is the causative agent of histomonosis in gallinaceous birds, predominantly in turkeys and chickens. Depending on the host species the outcome of the disease can be very severe with high mortality as observed in turkeys, whereas in chickens the mortality rates are generally lower. The disease is known for more than 100 years when *in vitro* and *in vivo* investigations started to understand histomonosis and the causative pathogen. For decades histomonosis could be well-controlled by effective drugs for prevention and therapy until the withdrawal of such chemicals for reasons of consumer protection in Europe, the USA and additional countries worldwide. Consequently, research efforts also focused to find new strategies against the disease, resulting in the development of an efficacious live-attenuated vaccine. In addition to efficacy and safety several studies were performed to obtain a deeper understanding of the immune response of the host against *H. meleagridis*. It could be demonstrated that antibodies accumulate in different parts of the intestine of chickens following infection with *H. meleagridis* which was much pronounced in the ceca. Furthermore, expression profiles of various cytokines revealed that chickens mounted an effective cecal innate immune response during histomonosis compared to turkeys. Studying the cellular immune response following infection and/or vaccination of host birds showed a limitation of pronounced changes of B cells and T-cell subsets in vaccinated birds in comparison to non-protected birds. Additionally, numbers of lymphocytes including cytotoxic T cells increased in the ceca of diseased turkeys compared to infected chickens suggesting an immunopathological impact on disease pathogenesis. The identification of type 1 and type 2 T-helper (Th) cells in infected and lymphoid organs by *in situ* hybridization did not show a clear separation of Th cells during infection but revealed a coherence of an increase of interferon (IFN)-γ mRNA positive cells in ceca and protection. The present review not only summarizes the research performed on the immune response of host birds in the course of histomonosis but also highlights the specific features of *H. meleagridis* as a model organism to study immunological principles of an extracellular organism in birds.

## Introduction

*Histomonas meleagridis* is an important flagellated parasite of poultry causing the disease histomonosis (syn. blackhead disease, histomoniasis, or infectious typhlohepatitis) (1). Historically, the disease was extensively investigated in the first half of the last century and thereby effective chemotherapeutics were identified to prevent and treat birds from infection. This success neglects that for a long time the true etiology of the disease was questioned and under debate. Difficulties to determine the real cause of histomonosis in earlier studies are comprehensively recapitulated elsewhere (2). However, to date the disease is of high relevance in poultry flocks as effective prophylactic and therapeutic options are not available anymore in many countries for reasons of food safety. As a consequence research was intensified in recent years and with it several reviews were published addressing different features of the parasite or the disease. This includes a general overview on the disease (3), updated findings of the recent years (4), a summary of experimental infections (5), a recapitulation on previous and current strategies for prevention and therapy (6), and assumptions how the disease might be controlled in the future (7).

The purpose of this review is to emphasize on studies investigating mechanisms of the immune response of host birds against the disease. This includes early studies describing inflammatory reactions of birds' up to recent investigations on specific immune cells and signaling proteins involved in host defense. Furthermore, the host reaction due to vaccination and its functional aspects are reviewed. Finally, *H. meleagridis* might be a model to unravel peculiar immune mechanisms of extracellular pathogens considering that the avian immune response against these organisms is not as investigated in depth compared to viral or bacterial infections.

### Histomonosis, an important poultry disease

Histomonosis was firstly described in turkeys by Cushman (8) more than a century ago. Infection with *H. meleagridis* can occur directly or via embryonated eggs of the nematode *Heterakis gallinarum* which was already described by Graybill and Smith (9). Horizontal transmission was hypothesized to occur by active uptake via the cloaca (10) or orally, based on successful oral application of cultured histomonads (11).The first signs of histomonosis are reflected by clinical changes such as reduced appetite, depression, drowsiness, droopy wings, and ruffled feathers. Infected birds might suffer from yellowish diarrhea and succumb to death (4). The pathogenesis generally varies between species of gallinaceous birds: in turkeys (*Meleagris gallopavo*) the disease can cause high mortality due to severe necrotic inflammation of the ceca and the liver, while in chickens (*Gallus gallus*) clinical signs are milder and pathological manifestations are often restricted to the ceca of infected birds.

Following infection, *H. meleagridis* migrates into the mucosa and deeper layers of the cecal wall leading to inflammation and ulceration, resulting in a thickening of the cecal tissue and formation of fibrin. Occasionally, ulcers erode throughout the cecal wall leading to peritonitis. Following destruction of cecal tissue, the parasite is able to infiltrate into blood vessels and to reach the liver via the portal vein. As a consequence, areas of inflammation and necrosis can occur in the liver. Liver lesions are highly variable in appearance: they may be up to 4 cm in diameter and can involve parts or the entire organ. Liver and cecal lesions together are a strong hint during post mortem investigations. The disease causes generally less severe lesions in chickens. Especially changes in the liver occur less frequently in chickens as compared to turkeys. In the final stage, the disease may become systemic when DNA of histomonads can be found in the blood and in the tissues of many organs, whether lesions are present or not (12). Lesions can be observed in different organs beside cecum and liver, such as kidneys, bursa of Fabricius, spleen, and pancreas (13–15). Apart from turkeys and chickens, other members of the galliformes, including pheasants, partridges, and farm-reared bobwhite quails can serve as hosts (16–19). In contrary, other avian species like ostriches and ducks show a high resistance to disease even though they may contribute to the transmission of the parasite (20, 21).

### *Histomonas meleagridis*, a unique protozoan parasite

*H. meleagridis* is a member of the family *Dientamoebidae*, order Tritrichomonadida (22). The parasite mainly possesses cell organelles that are typical for trichomonads (3). It is pleomorphic and generally two forms of the parasite are known: (i) the tissue form and (ii) the cecal lumen dwelling form. The tissue form is almost round with 6–20 μm in size and capable of forming pseudopodia (23, 24). Unlike the tissue form the cecal lumen form (3–16 μm) has a single flagellum although early during cell division, two may be observed (25). It was observed that the flagellum is getting lost during the invasion in the host tissue (26). In culture, *H. meleagridis* exhibits the morphology of the lumen-dwelling form. More recently, the occurrence of a cyst-like stage was reported (27). Later on, this resistant stage of *H. meleagridis* was investigated *in vitro* and it could be observed independent of the passage level and pathogenicity *in vivo* indicating an early adaption to *in vitro* conditions (28).

*H. meleagridis* is antigenetically (29) closely related to the intestinal parasite *Dientamoeba fragilis*, a trichomonad with a wider host range in mammals which is suspected to be associated with gastrointestinal disorders in humans. *Dientamoeba fragilis* is a protozoan parasite often described as “neglected parasite” (30). Recently, several major advances have been made with respect to this organism's life cycle and molecular biology, although knowledge on immune response against the pathogen is scant. The pathogenic potential of *D. fragilis* is still debatable. However, because of the close relativity to histomonads, the immunological research on *H. meleagridis* can give an indication to the immunological responsiveness of host against *D. fragilis*.

Hyperimmune antisera raised in rabbits against the two flagellates cross-reacted in an indirect fluorescent antibody test (31), although in agar gel immune-diffusion test (32) species-specific precipitin lines were seen. Both, antigenic differences and some cross-reactivity could also be demonstrated by immunoelectrophoresis (33). The nucleotide sequence analysis of a small subunit rRNA of the organism showed a close relationship between *D. fragilis* and *H. meleagridis* (34). First investigations on specific proteins of *H. meleagridis* were performed by Mazet et al. (35). The authors characterized genes encoding three proteins involved in hydrogenosomal carbon metabolism: a nicotinamide adenine dinucleotide phosphate-dependent hydrogenosomal malic enzyme, an α-subunit of a succinyl coenzyme-A synthetase and an iron-only hydrogenase. Afterwards, Bilic et al. (36) identified a broad spectrum of partial protein-coding sequences with homology to both intracellular and surface proteins. The antigenic potential of α-actinins of the parasite in host animals was later on demonstrated (37). Lynn and Beckstead, (38) applied splinkerette PCR to identify new genes. Their sequence analysis identified the 5′ coding portions of the β-tubulin genes, the intergenic regions, and two different open reading frames encoding for a putative serine/threonine phosphatase and a putative ras-related protein, racG. They predicted that these intergenic regions contain polyadenylation and cleavage signals for the two open reading frames and initiator elements for the β-tubulin genes. These regulatory elements are necessary for gene transcription in *H. meleagridis*. Most recently, sequencing of a cDNA library reported sequences of 3425 *H. meleagridis* genes (39). These analyses identified 81 genes coding for putative hydrogenosomal proteins and determined the codon usage frequency. That study also suggested that *H. meleagridis* α-actinins strongly contribute to the immune-reaction of host birds. Recently, *de novo* transcriptome sequencing of a virulent and an attenuated *H. meleagridis* strain provided novel insights into the parasite's biological processes, such as metabolism, locomotion, cell signaling and its ability to adapt to dynamic environmental changes (40). In addition, the study elucidated potential pathogenic mechanisms in respect to cytoadherence and host cell membrane disruption, together with the possible regulation of such processes. Monoyios et al. (41) addressed differences between *in vivo* cultivated virulent and attenuated *H. meleagridis* parasites on protein expression level. Based on mass spectrometry data it could be shown that eight different proteins, with the majority related to cellular stress management, have been found up-regulated in virulent histomonads compared to the attenuated strain which potentially affect the host-pathogen interaction between the two strains. Additionally, a virulence factor named legumain cysteine peptidase was detected. Applying two-dimensional electrophoresis in combination with mass spectrometric analysis 32 spots were identified as specific for the attenuated strain. These spots were described to correspond to the increased metabolism due to *in vitro* adaptation of the parasite and the amoeboid morphology.

## Immunological responses against histomonosis

Modulations of the innate and adaptive immune responses of the host by pathogens are known to be major determinants in the outcome of certain infectious diseases. Histomonosis causes severe disease in turkeys whereas less clinical signs occur in chickens as described above. This outcome can be linked with the host defense, indicating substantial differences between these two phylogenetically closely related species against *H. meleagridis*. Elucidating these differences in host response does not only unravel a certain host reaction it is also useful to understand protection and susceptibility in a broader context. Important studies investigating distinct parameter of the immune response against *H. meleagridis* are listed in Table 1.

**Table 1:** Year wise experimental studies in turkeys and/or chickens investigating important immunological parameters of histomonosis.

**Parameter**	**Components**	**Technique**	**Tissue**	**Host**	**Strain or antigenic preparation**	**Year of publication (reference)**
Serum antibodies	Precipitating antibodies	Agar gel immunodiffusion (Ouchterlony test)	Serum	Turkeys (Beltsville White) and chicken (Light Sussex cockerels)	Homogenate of cecum and liver tissues harvested from an infected turkey or ceca contents of an infected chicken	1963 (42)
Serum antibodies	IgG	Indirect immunofluorescence assay	Serum	Turkeys (breed BIG 6)	Virulent *H. meleagridis* (strain mdc), and a lysed (by sonication) preparation of the same strain	2009 (43)
Serum antibodies	IgG	indirect sandwich ELISA	Serum	Turkeys and chickens	Virulent *H. meleagridis*Turkey/Austria/2922-C6/04 or the same clones passaged for 95, 215 or 295 times	2009 (44)
Chemokine and cytokine mRNA	IL-1β, IL-6, CXCLi2, IL-10, TGF-β4, IFN-γ, IL-4, and IL-13	RT-qPCR	Cecal tonsil, liver and spleen	Turkeys and chickens (broilers and breeder cockerels)	A suspension of severely affected cecum and liver tissue homogenate harvested from chickens orally inoculated with embryonated eggs of *H. gallinarum*	2009 (45)
β-defensin mRNA	AvBD2		Cecal tonsil and liver			
Immune cells	CD4^+^, CD8α^+^, CD28^+^ and CD44^+^ cells	Immunohistochemistry	Liver and spleen			
Serum & mucosal antibodies	IgA, IgG, IgM	Indirect sandwich ELISA	Serum, duodenum, jejunum & cecum	Chickens	Clonal cultures of virulent (passaged for 21 times) *H. meleagridis* /Turkey/Austria/2922-C6/04	2010 (46)
Serum antibodies	IgG	Blocking ELISA	Serum	Turkeys (BUT 6) and chickens (Isa Brown layers)	A Dutch field strain (strain /Deventer/NL/AL327-type I/03)	2010 (47)
Serum antibodies	IgG	Indirect sandwich ELISA	Serum	Turkeys (BUT 9)	Clonal cultures of virulent (passaged for 21 times) and/or attenuated (passaged for 295 times) *H. meleagridis* / Turkey/Austria/2922-C6/04	2010 (48)
Serum antibodies	IgG	Indirect sandwich ELISA	Serum	Chickens (layer type)	Clonal cultures of virulent (passaged for 21 times) and/or attenuated (passaged for 295 times) *H. meleagridis* / Turkey/Austria/2922-C6/04	2013 (49)
Serum antibodies	IgG	indirect sandwich ELISA	Serum	Turkeys	Clonal cultures of attenuated (passaged for 295) *H. meleagridis* /Turkey/Austria/2922-C6/04 or the same strain back passaged *in vivo*	2013 (50)
Serum antibodies	IgG	Indirect sandwich ELISA	Serum	Chickens (layer and meat-type)	*H. meleagridis* strain Turkey/Germany/GB551/04 from an outbreak in a commercial meat turkey flock in Germany	2014 (51)
Immune cells	CD4^+^, CD8α^+^, B cells, heterophils, macrophages	Flow cytometry	cecum, liver, spleen, PBMC	Turkeys and chickens	Clonal cultures of virulent (passaged for 21 times) and/or attenuated (passaged for 295 times) *H. meleagridis*/Turkey/Austria/2922-C6/04	2017 (52)
	Heterophils, macrophages		Whole blood	Chickens		
Immune cells	T cells, B cells and monocytes/macrophages	Immunofluorescence	Cecum, liver, and spleen	Chickens	Clonal cultures of virulent (passaged for 21 times) and/or attenuated (passaged for 295 times) *H. meleagridis* /Turkey/Austria/2922-C6/04	2018 (53)
Type1/type2 signature cytokines	IFN-γ or IL-13 mRNA positive cells	*In situ* hybridization		Turkeys and chickens	

### Innate immune response

The first arm of the innate immune system against histomonosis is the anatomical barrier in the gastrointestinal tract. The parasite can infect its host via cloacal or oral route. However, oral inoculation was not always successful probably due to the acidity of the gizzard (10, 11, 54–56). The acid environment in the gizzard is a physiological barrier against pathogens and it was reported earlier that an effective infection depends upon the pH of the gizzard and the upper intestine (55). In the last mentioned work it was observed that the severity of lesions increased in chickens that have starved or were fed with an alkali mixture before the oral infection. Feed restriction after the application of live histomonads was shown to be an additional parameter which should be considered in the context of a successful oral infection (11).

Concerning the innate cellular response, first observations were made by histopathology in birds infected with *H. gallinarum* and *H. meleagridis* (57). Thereby, larvae of the cecal worm and an influx of heterophilic granulocytes were visible already from day 1 post infection (p.i.), even though first histomonads were only visualized after 5 days p.i. First lesions in the liver, characterized by lymphocytic infiltration with few heterophils at the portal area, were observed at the same time point (13). Specific detection of the parasite in tissue sections was described to be accompanied with infiltrations of mononuclear and polymorphonuclear cells in the infected organs cecum and liver (58, 59). In recent studies, quantitative analyses using specific markers against chicken macrophages/monocytes revealed that significantly higher amounts of this cell population were present in the blood (52) and the cecum (53) of infected chickens from the early stage of infection until the time period when most severe lesions were observed. The ability of macrophages to incorporate cells by phagocytosis indicates efforts to contain the parasite during the initial stage of infection in chickens. Furthermore, a lower presence of heterophils in the infected chickens' blood can be explained by the infiltration of these granulocytes to the local site of infection (52). Due to the lack of specific or cross-reactive antibodies for innate immune cells of turkeys it was so far not possible to generate comparative data in this more affected host species.

To investigate the innate cell signaling following infection, mRNA expression of the pro-inflammatory innate cytokines IL-1beta, IL-6, and CXCLi2 were measured in chickens and in turkeys after infection with histomonads (45). It was found that the immune response in the chicken was initiated in the cecal tonsils already after 1 day p.i. Interestingly, mRNA expression levels of these pro-inflammatory cytokines in turkeys were not up-regulated locally during the initial phase of infection even until the protozoa were already detectable in the liver. This depicts that an initial induced innate inflammatory response in the cecal tonsils may be critical to limit the dissemination of the parasite to the liver, with consequences on the clinical outcome within the different poultry species.

### Adaptive immune response

#### Pathogen-specific antibodies

The first study on specific antibodies against *H. meleagridis* was reported by Clarkson (42), who detected serum precipitins 7 days after infection. The attempt to transfer protective immunity by injections of serum from infected turkeys to naïve birds failed in the last mentioned study. Several years later, Powell et al. (45), observed increased antibody levels in sera of infected chickens compared to infected turkeys, but no further information on the methodology was given. More recently, vaccination against histomonosis using killed vaccines which elicit a dominantly antibody-mediated immune response was shown to be ineffective in providing protection (60). Similarly, Bleyen et al. (43) confirmed the inadequacy of serum antibodies in protecting turkeys from histomonosis, although the same immune component was shown to induce complement-mediated lysis of *H. meleagridis in vitro*. In recent years, an indirect sandwich ELISA (44), as well as a blocking ELISA using monoclonal antibodies (47) for the detection of antibodies against histomonads have been established. In these studies, an increase of antibodies in sera could be demonstrated in experimentally infected chickens and turkeys. Field studies on the prevalence of histomonads-specific antibodies in chicken flocks revealed a wide dissemination of the parasite in European countries (61, 62). In experimental studies, it was demonstrated that pathogen-specific serum antibodies increased already 2 weeks p.i. (44) and 3 weeks post vaccination with attenuated parasites above the cut off value until the following 13 weeks when the experiment was finished (48).

In a single study, the occurrence of different types of systemic and intestinal antibodies of chickens following infection with *H. meleagridis* was investigated by ELISA (46). Thereby, first optical density values for IgG above the cut-off in the serum were detected at 14 days p.i., whereas IgA and IgM levels remained low. Furthermore, it could be revealed that the intestinal tissue showed an intense humoral response in the parasitized ceca with an initial peak of IgM, high levels of IgG as well as a continuous increase of IgA and similar high levels of IgG together with IgA in the small intestine. Unfortunately, comparative results to the last mentioned studies in turkeys are not available which might be due to the lack of suitable reagents. However, along with an elevated level of antibodies the numbers of B cells increased in infected organs and systemically during infection were also reported recently in chickens and turkeys (52), which is outlined in the following chapter.

Another study, involving different lines of chickens, reported that antibody production differ due to the genetic background of the host (51). The study reported that the humoral immune response against actinin 1 started sooner and was significantly more pronounced in layer-type chickens than in meat-type chickens.

#### Cell-mediated immune response

First investigations on leukocytes were based on histopathology and indicated an influx of different populations of immune cells including lymphocytes in the infected organs cecum and liver (57). However, until recently there was no detailed information on the phenotype of immune cells that are involved in an adapted immune response and the link with the appearance following infection. In the last few years different studies were performed to investigate the mechanisms of the cellular modulation by detailed characterization of the involved leukocytes as well as cytokines triggering specific changes in the cellular response.

In general, the polarization of CD4^+^ T-helper (Th) and CD8^+^ T-cytotoxic (Tc) cells plays a major role in host-pathogen interaction. CD3^+^CD4^+^CD8α^−^ T cells are predominantly of helper phenotype, act as coordinators of the immune response by producing a variety of cytokines and secrete soluble molecules to the extracellular space which affects other cells of the immune system. In contrast, CD3^+^CD4^−^CD8α^+^ T cells are cytotoxic cells, promoting the cytolytic pathway. A protective immune response may rely on the ability of CD4^+^ T cells to accumulate high numbers of effector cells in order to activate a response against an invading pathogen. They can promote B cell-immunity with antibody production or, on the opposite, directly modulate, respectively control, the activity of different types of T cells. Secreted cytokines can activate macrophages and other cells through cell to cell signal communication. Powell et al. (45) used immunohistochemical stainings to specifically detect CD4^+^, CD8α^+^, CD28^+^, and CD44^+^ cells in the spleen as well as liver of chickens and turkeys infected with *H. meleagridis*. With this, they noticed an influx of the mentioned T cell-subpopulations into the liver of turkeys and chickens in coincidence with parasite infiltration. These cellular changes were more pronounced in turkeys and correlated with a decrease in numbers of such cells in spleens whereas no obvious changes were observed in the spleen of chickens.

By investigating T-cell subsets of chickens co-infected with *H. gallinarum* and *H. meleagridis*, a decrease of splenic CD4^+^ T cells together with a destruction of the cecal mucosa in association with a severe T cell infiltration in the cecal lamina propria was described (63).

In a more recent work, different populations of lymphocytes of host birds were analyzed by flow cytometry after vaccination with attenuated histomonads and/or infection using virulent parasites (52). Thereby, a detailed investigation on the adaptive immune system by investigating quantitative changes of CD4^+^, CD8α^+^ T cells and B cells in different organs and blood of turkeys and chickens was performed. In that study, all infected turkeys died by 14 days p.i. due to severe histomonosis whereas infected chickens or vaccinated birds were not clinically affected. It was hypothesized that the excessive necrosis of caecum and liver in infected tissues of turkeys might be an effect of cytotoxic activity of effector CD8^+^ T cells which still needs to be verified. The predominance of CD8α^+^ T cells might contribute to the destruction of the host tissue and the local suppression of other immune responses including the inhibition of CD4^+^ T-cell proliferation (52). This is supported by the finding that CD4^+^ T cells were significantly decreased in the cecum of infected turkeys. On the other hand, the challenge of vaccinated turkeys led to a significant increase of CD4^+^, CD8α^+^, and B cells in the blood already at 4 days post inoculation, indicating an effective and fast recall response of the primed immune system. In infected chickens the analyzed immune cells in cecum and liver were mostly in the range of values of non-infected birds matching with the lower lesion scores. However, a continuing recruitment of CD4^+^ and CD8α^+^ T cells was observed in the blood of infected chickens. Beside the translocation of these cells to the target organs of infection, this finding might also be explained by the presence of the parasite in the blood of infected host birds (14, 64, 65). In vaccinated as well as vaccinated and challenged chickens, changes of cecal B cells, CD4^+^ and CD8α^+^ T cells were in general even lower compared to infected chickens (52). Overall, such findings demonstrated that vaccination of turkeys and chickens using clonal cultures of *H. meleagridis* limits severe changes of B cells and T cell-subsets as compared to the exacerbated influx observed in non-protected animals. Additionally, a more intense cellular immune response in infected organs of turkeys in comparison to chickens was concluded to contribute to the fatal clinical outcome of the infection in turkeys.

Immunofluorescence and quantification of lymphocyte populations by image analyses, confirmed an influx of B cells and T cells in the infected chicken's cecum from 4 days p.i. until 10 days p.i. (53). In contrast, chickens that were vaccinated showed a similar range of the above mentioned cell population in the cecum compared to control birds even after challenge. Comparative data on turkey ceca obtained by immunofluorescence have so far not been reported due to the lack of cross-reactivity of those antibodies for this host species (52).

Investigations on cytokines in context of an immune response against *H. meleagridis* were performed in different studies by gene expression analyses and *in situ* hybridization for the detection of cells that contain transcripts of specific cytokines. Along with innate pro-inflammatory cytokines mentioned above, Powell et al. (45) investigated adaptive response-signature cytokines IFN-γ, IL-13, and IL-4 and the regulatory cytokines IL-10 and TGF-β4 by RT-qPCR in different organs of infected chickens and turkeys. Most important, in chickens, IFN-γ and IL-13 mRNA expression was up-regulated while IL-4 mRNA expression remained unaltered during infection. Expression of the regulatory cytokine IL-10 was up-regulated very early during infection in this host species while TGF-β4 mRNA expression levels were unchanged during the experiment. In turkeys, IFN-γ mRNA expression levels were down-regulated in the cecal tonsils soon after infection but up-regulated during later stages. IL-4 mRNA expression levels were variable while IL-13 again showed a sustained up-regulation. As in chickens, IL-10 did not appear to play a significant role during infection in turkeys, but TGF-β4 mRNA expression levels were increased.

Later on, Schwarz et al. (63) found a significant increase in mRNA expression of IFN-γ in chicken cecal tissue infected with *H. gallinarum* harboring histomonads in contrast to an elevated expression of IL-13 when chickens were infected only with *H. gallinarum*. The authors hypothesized that the IFN-γ over-expression in the co-infection was modulated by the presence of *H. meleagridis*. Nevertheless, based on the experimental setting it is difficult to determine if both parasites together cause a variant immune response.

Recently, Kidane et al. (53) investigated the abundance of Th1 and Th2 cytokines, IFN-γ, respectively IL-13 mRNA positive cells by *in situ* hybridization in vaccinated and/or infected chickens and turkeys. It was demonstrated that changes in the abundance of positive cells following infection or vaccination were less pronounced in chickens compared to turkeys. Infected turkeys showed an early decrease of cytokine mRNA positive cells in cecum which later increased together with a severe destruction of the mucosa and infiltration of cytokine expressing cells up to the muscularis layer. A similar destruction and cytokine distribution was observed in the liver of these birds. In comparison, an increased percentage of IFN-γ mRNA positive cells were noticed in vaccinated and challenged turkeys already 4 days post challenge confirming the priming of an immune response by vaccination. An interesting finding was that IFN-γ mRNA positive cells in the cecum of naïve chickens were distinctly higher than in naïve turkeys. These findings led to the conclusion that IFN-γ positive cells may act as a protective trait against histomonosis. However, no distinct Th1/Th2 separation in the immune response was noticed, indicating a more balanced activation of the Th pathways during infection with an extracellular protozoan parasite in birds. Moreover, it could be demonstrated that the fatal clinical outcome of turkeys due to histomonosis is in coherence with a more intense adaptive immune response in infected organs compared to chickens.

## Conclusion and outlook

The reviewed studies are fundamental in devising prospective immunoprophylactic strategies against histomonosis. Results on different types of vaccine either killed or live, revealed a possible direction into how a vaccine could successfully mount a protective immune response. Furthermore, it is crucial to understand relevant protective traits as well as the failure of the immune system against an infection with *H. meleagridis*.

**Figure 1:**
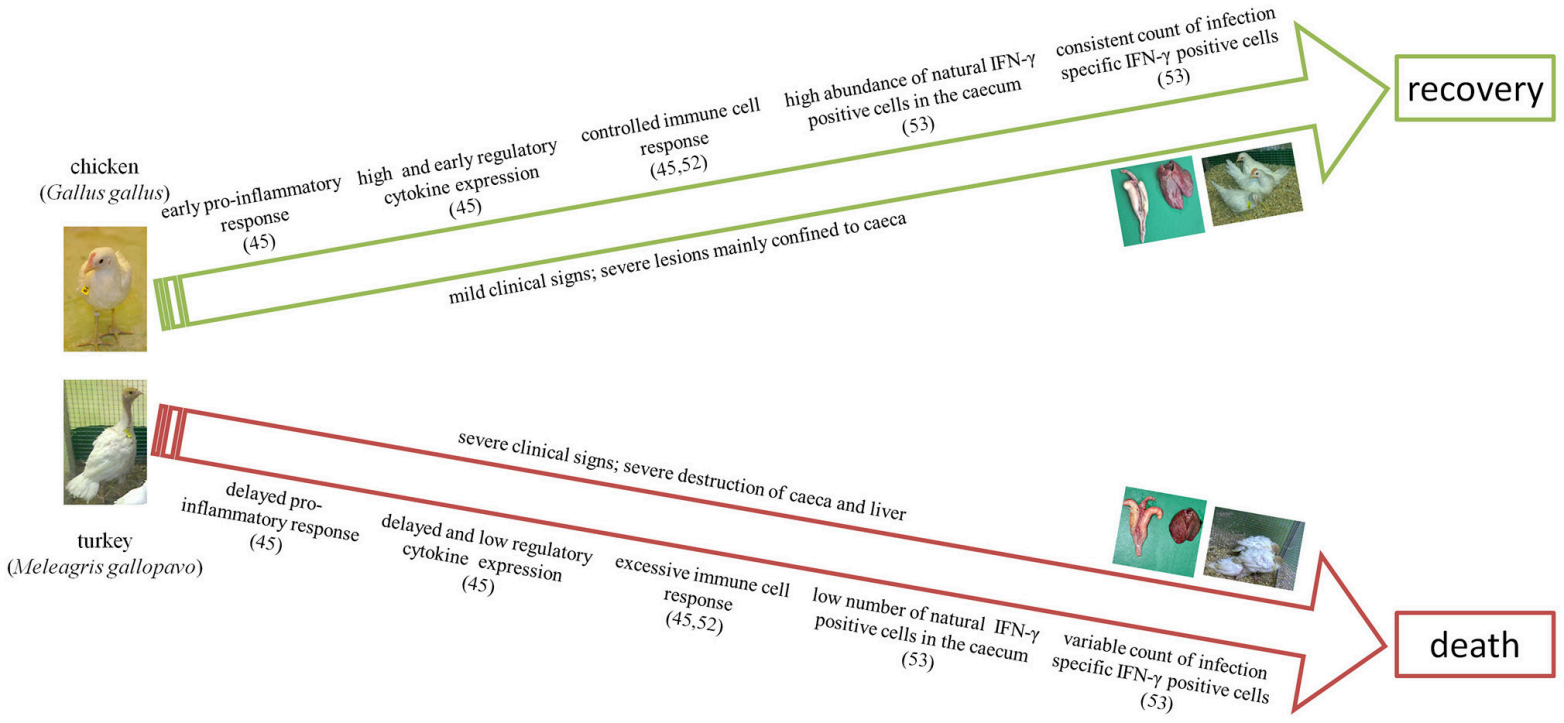
Key differences in the disease and immune response of the two main avian host species during infection with *H. meleagridis*. The numbers refer to the respective references.

The most peculiar and differing changes in the immune response in chickens and turkeys against histomonosis are drafted in Figure 1. From the experimental studies on the immune response during histomonosis we can clearly elicited that differing profiles of cytokine expression and abundances of specific immune cells resulted in a varying disease progression and outcome in the two main avian host species, chickens and turkeys. At the early infection phase chickens show an expeditious immune response against the parasite which triggers the immune cascade to restrict the parasite progression. In comparison, the turkey's immune responsiveness is delayed, which obviously allows the parasite to disseminate systematically to the liver and other organs. After the initial phase, the effectiveness of the adaptive immune response is based on the accessibility of natural IFN-γ positive cells and a controlled expression of adaptive immune cells which seem to be further key factors to minimize clinical signs and to induce the recovery of chickens. In contrast, a predominance of the cellular response toward the cytolytic pathway may be involved in aggravating tissue destruction in turkeys. Thus, un-controlled immune response and excessive destruction of the tissue can be understood as a further failure of the immune system with consequences on the fatal outcome of the disease in turkeys. Conclusions on the different immune response in chickens and turkeys are supported by the fact that vaccination triggered a similar enhanced allocation of IFN-γ cells and controlled adaptive cell response for both host species. Overall, it can be concluded that an early and locally induced immune response is the crucial factor behind the survival of chickens and immunoprophylaxis induced by vaccination independent of the host.

Further studies on the immune response of poultry against *Histomonas meleagridis* should consider both host and pathogen factors. Given the fact that turkeys and chickens display a different involvement of the immune response to *H. meleagridis*, it could be beneficial to use these contrasting host features in further exploring traits of the immune response. So far, there is hardly any information on the innate immune response against histomonosis. Especially the role of toll-like receptors (TLRs), with possible consequences on modulation of the immune response following vaccination and/or infection, needs to be understood. Furthermore, mechanisms on the function of innate immunity, particularly pro-inflammatory cytokines and antigen-presenting cells, could be useful to link the transition from the innate to the adaptive stage of the immune response. This can unveil essential features such as the quality and persistence of the acquired immune response which is helpful in establishing vaccination schedules. Data collected in experimental studies investigating histomonosis or following vaccination against the disease revealed important changes in the immune response but further identification on pathogen-specific mechanisms would be valuable. Hence, determining specific immunological correlates of protection e.g., the role and function of pathogen-specific T cells would contribute in pin-pointing features that mediate protection. Consequently, unraveling selective mechanisms that induce protection would be useful to promote such effector functions for facilitating new prospects in research on vaccination against histomonosis. Finally, along with studies on screening virulence factors of the protozoa, further explorations on the molecular plethora for potential immunogenic components are necessary to explain pathogen-directed immune reactions of the host.

## Author contributions

TM, FK, MH, and DL conceived and designed the review. All authors read and approved the final manuscript.

### Conflict of interest statement

The authors declare that the research was conducted in the absence of any commercial or financial relationships that could be construed as a potential conflict of interest.

## References

[1] Smith T An infectious disease among turkeys caused by protozoa (infectious entero-hepatitis). USDA Bur Anim Ind Bull. (1895) 8:3–27.

[2] Hess M. Commensal or pathogen—A challenge to fulfil Koch‘s Postulates. Br Poult Sci. (2017) 58:1–12. 10.1080/00071668.2016.1245849 27724044 PMC5359748

[3] McDougald LR. Blackhead disease (histomoniasis) in poultry: a critical review. Avian Dis. (2005) 49:462–76. 10.1637/7420-081005R.1 16404985

[4] Hess M Liebhart D Bilic I Ganas P. *Histomonas meleagridis*—New insights into an old pathogen. Vet Parasitol. (2015) 208:67–76. 10.1016/j.vetpar.2014.12.018 25576442

[5] Hauck R Hafez HM. Experimental infections with the protozoan parasite *Histomonas meleagridis*: a review. Parasitol Res. (2013) 112:19–34. 10.1007/s00436-012-3190-5 23160894

[6] Liebhart D Ganas P Sulejmanovic T Hess M. Histomonosis in poultry: previous and current strategies for prevention and therapy. Avian Pathol. (2017) 46:1–18. 10.1080/03079457.2016.1229458 27624771

[7] Clark S Kimminau E. Critical review: future control of blackhead disease (histomoniasis) in poultry. Avian Dis. (2017) 61:281–8. 10.1637/11593-012517-ReviewR 28957000

[8] Cushman S The Production of Turkeys. Kingston, RI: Bulletin 25, Agricultural Experiment Station, Rhode Island College of Agriculture and Mechanical Arts (1893). 89–123.

[9] Graybill HW Smith T. Production of fatal blackhead in turkeys by feeding embryonated eggs of *Heterakis papillosa*. J Exp Med. (1920) 31:647–55. 10.1084/jem.31.5.647 19868419 PMC2128241

[10] Hu J Fuller L McDougald LR. Infection of turkeys with *Histomonas meleagridis* by the cloacal drop method. Avian Dis. (2004) 48:746–50. 10.1637/7152 15666855

[11] Liebhart D Hess M. Oral infection of turkeys with *in vitro*-cultured *Histomonas meleagridis* results in high mortality. Avian Pathol. (2009) 38:223–7. 10.1080/03079450902912192 19468939

[12] Huber K Reynaud M-C Callait MP Zenner L. *Histomonas meleagridis* in turkeys: dissemination kinetics in host tissues after cloacal infection. Poult Sci. (2006) 85:1008–14. 10.1093/ps/85.6.1008 16776468

[13] Malewitz TD. The pathology of experimentally produced histomoniasis in turkeys. Am J Vet Res. (1958) 19:181–5. 13498258

[14] McGuire WC Morehouse NF. Blood-induced blackhead. J Parasitol. (1958) 44:292–6. 13550023

[15] Peardon DL Ware JE. Atypical foci of histomoniasis lesions in a study of direct oral transmission. Avian Dis. (1969) 13:340–4. 10.2307/1588502 5816046

[16] Potts GR. Long-term changes in the prevalences of caecal nematodes and histomonosis in gamebirds in the UK and the interaction with poultry. Vet Rec. (2009) 164:715–8. 10.1136/vr.164.23.715 19502627

[17] Reis JL Jr Beckstead RB Brown CC Gerhold RW. *Histomonas meleagridis* and capillarid infection in a captive chukar (*Alectoris chukar*). Avian Dis. (2009) 53:637–9. 10.1637/8678-030409-Case.1 20095170

[18] McDougald LR Abraham M Beckstead RB. An outbreak of blackhead disease (*Histomonas meleagridis*) in farm-reared bobwhite quail (*Colinus virginianus*). Avian Dis. (2012) 56:754–6. 10.1637/10140-032212-Case.1 23397851

[19] Liebhart D Neale S Garcia-Rueda C Wood AM Bilic I Wernsdorf P. A single strain of *Tetratrichomonas gallinarum* causes fatal typhlohepatitis in red-legged partridges (*Alectoris rufa*) to be distinguished from histomonosis. Avian Pathol. (2014) 43:473–80. 10.1080/03079457.2014.959435 25175532

[20] Gordo FP Herrera S Castro AT Durán BG Diaz RAM Parasites from farmed ostriches (*Struthio camelus*) and rheas (*Rhea americana*) in Europe. Vet Parasitol. (2002) 107:137–60. 10.1016/S0304-4017(02)00104-8 12072221

[21] Callait-Cardinal MP Chauve C Reynaud MC Alogninouwa T Zenner L. Infectivity of *Histomonas meleagridis* in ducks. Avian Pathol. (2006) 35:109–16. 10.1080/03079450600597626 16595302

[22] Cepicka I Hampl V Kulda J. Critical taxonomic revision of parabasalids with description of one new genus and three new species. Protist (2010) 161:400–33. 10.1016/j.protis.2009.11.005 20093080

[23] Tyzzer EE. Developmental phases of the protozoon of “Blackhead” in turkeys. J Med Res. (1919) 40:1–30. 19972476 PMC2104353

[24] Tyzzer EE The flagellate character and reclassification of the parasite producing “Blackhead” in turkeys: *Histomonas* (gen. nov.) *meleagridis* (Smith). J Parasitol. (1920) 6:124–31. 10.2307/3271065

[25] Honigberg BM Benett CJ. Lightmicroscopic observations on structure and division of *Histomonas meleagridis* (Smith). J Eukaryot Microbiol. (1971) 18:687–97. 4943763 10.1111/j.1550-7408.1971.tb03398.x

[26] Bishop A Histomonas meleagridis in domestic fowls (*Gallus gallus*). Cultivation and experimental infection. Parasitology (1938) 30:181–94. 10.1017/S0031182000025749

[27] Zaragatzki E Hess M Grabensteiner E Abdel-Ghaffar F Al-Rasheid KAS Mehlhorn H. Light and transmission electron microscopic studies on the encystation of *Histomonas meleagridis*. Parasitol Res. (2010) 106:977–83. 10.1007/s00436-010-1777-2 20143091

[28] Gruber J Ganas P Hess M. Long-term *in vitro* cultivation of *Histomonas meleagridis* coincides with the dominance of a very distinct phenotype of the parasite exhibiting increased tenacity and improved cell yields. Parasitology (2017) 144:1253–63. 10.1017/S0031182017000646 28478784

[29] Dwyer DM. Analysis of the antigenic relationships among *Trichomonas, Histomonas, Dientamoeba*, and *Entamoeba*. I. Quantitative fluorescent antibody methods. J Eukaryot Microbiol. (1972) 19:316–25. 10.1111/j.1550-7408.1972.tb03467.x 4113358

[30] Stark D Barratt J Chan D Ellis JT *Dientamoeba fragilis*, the neglected trichomonad of the human bowel. Clin Microbiol. (2016) 29(3):553–80. 10.1128/CMR.00076-15 PMC4861990 27170141

[31] Dwyer DM Honigberg BM. Immunologic analysis by quantitative fluorescent antibody methods of effects of prolonged cultivation on *Histomonas meleagridis* (Smith). Z Parasitenkd (1972) 39:39–52. 4558134 10.1007/BF00329219

[32] Dwyer DM. Analysis of the antigenic relationships among *Trichomonas, Histomonas, Dientamoeba*, and *Entamoeba*. II. Gel diffusion methods. J Eukaryot Microbiol. (1972) 19:326–32. 10.1111/j.1550-7408.1972.tb03468.x 4624302

[33] Dwyer DM Analysis of the antigenic relationships among *Trichomonas, Histomonas, Dientamoeba*, and *Entamoeba* III. immunoelectrophoresis technics. J Eukaryot Microbiol. (1974) 21:139–45. 10.1111/j.1550-7408.1974.tb03628.x 4817975

[34] Gerbod D Edgcomb VP Noël C Zenner L Wintjens R Delgado-Viscogliosi P. Phylogenetic position of the trichomonad parasite of turkeys, *Histomonas meleagridis* (smith) tyzzer, inferred from small subunit rRNA sequence1. J Eukaryot Microbiol. (2005) 48:498–504. 10.1111/j.1550-7408.2001.tb00185.x 11456328

[35] Mazet M Diogon M Alderete JF Vivares CP Delbac F. First molecular characterisation of hydrogenosomes in the protozoan parasite *Histomonas meleagridis*. Int J Parasitol. (2008) 38:177–90. 10.1016/j.ijpara.2007.06.006 17697679

[36] Bilic I Leberl M Hess M. Identification and molecular characterization of numerous *Histomonas meleagridis* proteins using a cDNA library. Parasitology (2009) 136:379–91. 10.1017/S0031182008005477 19154645 PMC2957082

[37] Leberl M Hess M Bilic I. *Histomonas meleagridis* possesses three alpha-actinins immunogenic to its hosts. Mol Biochem Parasitol. (2010) 169:101–7. 10.1016/j.molbiopara.2009.10.007 19896981

[38] Lynn EC Beckstead RB. Identification of gene expression elements in *Histomonas meleagridis* using splinkerette PCR, a variation of ligated adaptor PCR. J Parasitol. (2012) 98:135–41. 10.1645/GE-2916.1 21864131

[39] Klodnicki ME McDougald LR Beckstead RB. A genomic analysis of *Histomonas meleagridis* through sequencing of a cDNA library. J Parasitol. (2013) 99:264–9. 10.1645/GE-3256.1 23075009

[40] Mazumdar R Endler L Monoyios A Hess M Bilic I. Establishment of a *de novo* reference transcriptome of *Histomonas meleagridis* reveals basic insights about biological functions and potential pathogenic mechanisms of the parasite. Protist (2017) 168:663–85. 10.1016/j.protis.2017.09.004 29107797

[41] Monoyios A Patzl M Schlosser S Hess M Bilic I. Unravelling the differences: comparative proteomic analysis of a clonal virulent and an attenuated *Histomonas meleagridis* strain. Int J Parasitol. (2018) 48:145–57. 10.1016/j.ijpara.2017.08.017 29203214

[42] Clarkson MJ. Immunological responses to *Histomonas meleagridis* in the turkey and fowl. Immunology (1963) 6:156–68. 14021587 PMC1423222

[43] Bleyen N Ons E De Gussem M Goddeeris BM Passive immunization against *Histomonas meleagridis* does not protect turkeys from an experimental infection. Avian Pathol. (2009) 38:71–6. 10.1080/03079450802641255 19156583

[44] Windisch M Hess M. Establishing an indirect sandwich enzyme-linked-immunosorbent-assay (ELISA) for the detection of antibodies against *Histomonas meleagridis* from experimentally infected specific pathogen-free chickens and turkeys. Vet Parasitol. (2009) 161:25–30. 10.1016/j.vetpar.2008.12.014 19162403 PMC2957077

[45] Powell FL Rothwell L Clarkson MJ Kaiser P. The turkey, compared to the chicken, fails to mount an effective early immune response to *Histomonas meleagridis* in the gut. Parasite Immunol. (2009) 31:312–27. 10.1111/j.1365-3024.2009.01113.x 19493211

[46] Windisch M Hess M. Experimental infection of chickens with *Histomonas meleagridis* confirms the presence of antibodies in different parts of the intestine. Parasite Immunol. (2010) 32:29–35. 10.1111/j.1365-3024.2009.01159.x 20042005

[47] van der Heijden HMJF Stegeman A Landman WJM. Development of a blocking-ELISA for the detection of antibodies against *Histomonas meleagridis* in chickens and turkeys. Vet Parasitol. (2010) 171:216–22. 10.1016/j.vetpar.2010.03.028 20400229

[48] Liebhart D Windisch M Hess M. Oral vaccination of 1-day-old turkeys with *in vitro* attenuated *Histomonas meleagridis* protects against histomonosis and has no negative effect on performance. Avian Pathol. (2010) 39:399–403. 10.1080/03079457.2010.506906 20954017

[49] Liebhart D Sulejmanovic T Grafl B Tichy A Hess M. Vaccination against histomonosis prevents a drop in egg production in layers following challenge. Avian Pathol. (2013) 42:79–84. 10.1080/03079457.2012.760841 23391185

[50] Sulejmanovic T Liebhart D Hess M *In vitro* attenuated *Histomonas meleagridis* does not revert to virulence, following serial *in vivo* passages in turkeys or chickens. Vaccine (2013) 31:5443–50. 10.1016/j.vaccine.2013.08.098 24055087

[51] Lotfi A Hauck R Olias P Hafez HM. Pathogenesis of histomonosis in experimentally infected specific-pathogen-free (SPF) layer-type chickens and SPF meat-type chickens. Avian Dis. (2014) 58:427–32. 10.1637/10782-012814-Reg.1 25518438

[52] Mitra T Gerner W Kidane FA Wernsdorf P Hess M Saalmüller A. Vaccination against histomonosis limits pronounced changes of B cells and T-cell subsets in turkeys and chickens. Vaccine (2017) 35:4184–96. 10.1016/j.vaccine.2017.06.035 28662952 PMC5604733

[53] Kidane FA Mitra T Wernsdorf P Hess M Liebhart D Allocation of interferon (IFN)-gamma mRNA positive cells in caecum hallmarks a protective trait against histomonosis. Front Immunol. (2018) 9:1164 10.3389/fimmu.2018.01164 29892298 PMC5985309

[54] Farmer RK Stephenson J Infectious enterohepatitis (blackhead) in turkeys; a comparative study of methods of infection. J Comp Pathol Ther. (1949) 59:119–26.

[55] Horton-Smith C Long PL. Studies in histomoniasis: I. The infection of chickens (*Gallus gallus*) with histomonad suspensions. Parasitology (1956) 46:79–90. 10.1017/S0031182000026354 13322457

[56] Lund EE Oral transmission of Histomonas in turkeys. Poult Sci. (1956) 35:900–4.

[57] Tyzzer EE A study of immunity produced by infection with attenuated culture-strains of *Histomonas meleagridis*. J Comp Pathol Ther. (1936) 49:285–303. 10.1016/S0368-1742(36)80025-3

[58] Liebhart D Grabensteiner E Hess M. A virulent mono-eukaryotic culture of *Histomonas meleagridis* is capable of inducing fatal histomonosis in different aged turkeys of both sexes, regardless of the infective dose. Avian Dis. (2008) 52:168–72. 10.1637/8107-090707-ResNote 18459318

[59] Singh A Weissenböck H Hess M. *Histomonas meleagridis*: immunohistochemical localization of parasitic cells in formalin-fixed, paraffin-embedded tissue sections of experimentally infected turkeys demonstrates the wide spread of the parasite in its host. Exp Parasitol. (2008) 118:505–13. 10.1016/j.exppara.2007.11.004 18155698

[60] Hess M Liebhart D Grabensteiner E Singh A. Cloned *Histomonas meleagridis* passaged *in vitro* resulted in reduced pathogenicity and is capable of protecting turkeys from histomonosis. Vaccine (2008) 26:4187–93. 10.1016/j.vaccine.2008.05.071 18586362

[61] Grafl B Liebhart D Windisch M Ibesich C Hess M. Seroprevalence of *Histomonas meleagridis* in pullets and laying hens determined by ELISA. Vet Rec. (2011) 168:160. 10.1136/vr.c6479 21493512

[62] van der Heijden HMJF Landman WJM. High seroprevalence of *Histomonas meleagridis* in Dutch layer chickens. Avian Dis. (2011) 55:324–7. 10.1637/9609-120610-ResNote.1 21793452

[63] Schwarz A Gauly M Abel H Daş G Humburg J Weiss ATA. Pathobiology of *Heterakis gallinarum* mono-infection and co-infection with *Histomonas meleagridis* in layer chickens. Avian Pathol. (2011) 40:277–87. 10.1080/03079457.2011.561280 21711187

[64] Clarkson MJ The progressive pathology of Heterakis-produced histomoniasis in turkeys. Res Vet Sci. (1962) 3:443–8.

[65] Farmer RK Hughes DL Whiting G. Infectious enterohepatitis (blackhead) in turkeys: a study of the pathology of the artificially induced disease. J Comp Pathol Ther. (1951) 61:251–62. 10.1016/S0368-1742(51)80025-0 14888729

